# Non–β-hemolytic Streptococcal Bacteremia in Patients With Heart Valve Prosthesis—Is It Always Infective Endocarditis?

**DOI:** 10.1093/ofid/ofaf242

**Published:** 2025-04-18

**Authors:** Fanni Toivonen, Torgny Sunnerhagen, Johannes Lundin, Anna Bläckberg, Sigurdur Ragnarsson, Magnus Rasmussen

**Affiliations:** Division of Infection Medicine, Department of Clinical Sciences, Lund, Lund University, Lund, Sweden; Division of Infection Medicine, Department of Clinical Sciences, Lund, Lund University, Lund, Sweden; Department of Clinical Microbiology, Infection Control and Prevention, Office for Medical Services, Region Skåne, Lund, Sweden; Division of Infection Medicine, Department of Clinical Sciences, Lund, Lund University, Lund, Sweden; Division of Infection Medicine, Department of Clinical Sciences, Lund, Lund University, Lund, Sweden; Department of Infectious Diseases, Skåne University Hospital, Lund, Sweden; Division of Thoracic Surgery, Department of Clinical Sciences, Lund, Lund University, Lund, Sweden; Department of Thoracic Surgery, Skåne University Hospital, Lund, Sweden; Division of Infection Medicine, Department of Clinical Sciences, Lund, Lund University, Lund, Sweden; Department of Infectious Diseases, Skåne University Hospital, Lund, Sweden

**Keywords:** bacteremia, endocarditis, heart valve prosthesis, streptococcus, viridans streptococci

## Abstract

**Background:**

Non–β-hemolytic streptococci are among the most common causative agents of infective endocarditis (IE). Patients with a heart valve prosthesis (HVP) have a high risk of IE. We aimed to determine the risk for IE in patients with HVP and non–β-hemolytic streptococcal bacteremia (NBHSB) and to study NBHSB relapse depending on treatment duration.

**Method:**

Adults with HVP and NBHSB from 2015 to 2018 in the region of Skåne, Sweden, were identified through the Clinical Microbiology Laboratory in Lund and evaluated in a population-based investigation. Data were collected from medical records according to a predefined protocol.

**Results:**

A total of 110 NBHSB episodes in 89 patients with HVP were included. In 40 episodes (36%), the patients had definite IE, in 69 possible IE, and 1 had rejected IE according to the European Society of Cardiology 2015 criteria. Of the 70 patients without a definite diagnosis of IE, 28 (40%) were treated with antibiotics as if they had IE. There were 7 NBHSB relapses, and 6 of these occurred in patients with possible IE. Four relapses occurred in patients who received antibiotics for <14 days. Three patients with possible IE were diagnosed with definite IE at the time of the relapse.

**Conclusions:**

Patients with NBHSB and HVP have a high risk for IE and should be thoroughly investigated. Most patients with NBHSB and HVP who fulfilled criteria for possible IE did not receive long-course antibiotic treatment. Moreover, some patients treated with a short course of antibiotics experienced NBHSB relapse.

## Teaser

Many patients with non–β-hemolytic streptococcal bacteremia and heart valve prosthesis had possible infective endocarditis, but most were not treated as such. Some had a relapse, suggesting inadequate treatment. These patients are at high risk of infective endocarditis, warranting thorough investigation.

Non–β-hemolytic streptococci, also referred to as oral or viridans streptococci, are among the most common causative agents of infective endocarditis (IE) [1], accounting for approximately 12% to 36% of cases [2–4]. It is well known that patients with a heart valve prosthesis (HVP) are more susceptible to develop IE, and it has been shown that HVP is an independent risk factor for IE in non–β-hemolytic streptococcal bacteremia (NBHSB) [5, 6]. Due to the increasing use of surgical valve replacement for the treatment of valvular heart disease, the number of patients at risk for IE is growing.

Traditionally, the Duke criteria have been used to guide the diagnosis and evaluate the likelihood of IE. First established in 1994, the Duke criteria have major and minor criteria that classify cases into definite, possible, or rejected IE by integrating microbiological, imaging, and clinical information [7]. The initial iteration of the Duke criteria incorporated specific echocardiographic findings to enhance the diagnostic accuracy of IE [7]. A diagnosis of definite IE requires the presence of 2 major criteria, 1 major criterion and 3 minor criteria, or 5 minor criteria; however, for possible IE, 1 major and 1 minor criterion or 3 minor criteria are enough. Although the Duke criteria have undergone modifications throughout the years, blood culture positivity remains the foundation of IE diagnosis [8–10].

The discovery of NBHSB is a reoccurring clinical scenario in which the risk for IE must be considered. At least 2 risk stratification systems can be used to evaluate if there is a need to perform echocardiography in patients with NBHSB. In both these systems, HVP is included as a risk factor [11, 12]. When IE is suspected in patients with HVP, transthoracic echocardiography (TTE) and transesophageal echocardiography (TOE) are recommended [13]. However, the sensitivity of echocardiography is not optimal in patients with HVP [14], with reported sensitivity of 33% to 70% for TTE and 86% to 92% for TOE [8, 15]. Thus, in patients with a high risk of IE and negative TOE, further investigations can be considered, including repeated TOE, computed tomography (CT) of the heart or other parts of the body, or positron emission tomography (PET)/CT [16].

Despite thorough investigation, many patients with NBHSB and HVP will have inconclusive imaging diagnostics and will not undergo surgery or postmortem examination to confirm IE. In the absence of an alternative diagnosis, these cases will typically be classified as possible IE, as they fulfill the minor criterion of predisposition and often the major criterion of blood culture positivity. Since there are no guidelines on how such patients should be managed and treated, we sought to describe this in a retrospective observational cohort study. The aim was to investigate factors associated with IE in patients with NBHSB and HVP and to study the frequency of NBHSB relapse depending on antibiotic treatment duration.

## MATERIALS AND METHODS

### Study Cohort

This retrospective, population-based, observational study was approved by the Regional Ethical Review Authority (2018/898). Patients with NBHSB between 2015 and 2018 in the region of Skåne, Sweden, were identified through the Clinical Microbiology Laboratory in Lund, Sweden. The laboratory serves all 10 hospitals in the region of Skåne. During the study period, BACTEC FX (Becton Dickinson) was used for blood culture, and MALDI TOF-MS (Bruker MBT Compass Library; Bruker Daltonics) was used for species determination. The medical records of patients aged ≥18 years were examined, and those with HVP at the time of NBHSB constituted the study population. Clinical data were collected from the medical records of these patients according to a predefined protocol. All NBHSB episodes were included in the analyses, as were those in patients with multiple episodes during the study period.

### Definitions

IE was defined according to the modified Duke criteria/European Society of Cardiology criteria from 2015 by Habib et al [8]. Cases that were classified as possible or rejected IE according to this set of criteria were considered nondefinite IE. Information was collected on comorbidities prior to the NBHSB episode. The comorbidities were classified according to the Charlson Comorbidity Index updated and validated by Quan et al [17]. Nosocomial NBHSB was defined as bloodstream infection occurring >48 hours after hospitalization. Health care–associated NBHSB was defined according to Friedman et al [18]. The HANDOC score was calculated for each episode according to the score system presented by Sunnerhagen et al [11]. Relapse was defined as the recurrence of bacteremia with the same species within 90 days after completion of antibiotic treatment. Data were collected on death within 30 days after a positive blood culture result. Sepsis was defined according to the Sepsis-3 criteria [19].

### Statistical Analysis

Categorical variables are reported as counts and percentages. Nonnormally distributed continuous variables are reported as median and IQR. For categorical variables, the Fisher exact test was performed. For the categorical variable Charlson Comorbidity Index score, the Pearson χ^2^ test was conducted. For nonnormally distributed continuous variables, the Mann-Whitney *U* test was performed.

To compare the different non–β-hemolytic streptococcal subgroups, each subgroup was used at a time and compared with the other groups combined as a reference group, with the Fisher exact test. Similarly, to compare the type of valve prosthesis, each type of valve prosthesis was used at a time and compared with the other types of valve prostheses combined as the reference group.

When the mode of acquisition of bacteremia was compared, all episodes of community-acquired and health care–associated NBHSB were combined and then compared with episodes of nosocomial NBHSB. To compare the location of prosthetic valve in the 2 groups, patients with an aortic valve prosthesis were compared with patients without one. Episodes without a focal infection were compared with episodes where a focal infection of any kind was identified.

For all the analyses performed, *P* < .05 was regarded as significant. All statistical analyses were performed in Stata version 18 (StataCorp).

## RESULTS

### Patient Population

**Figure 1.**
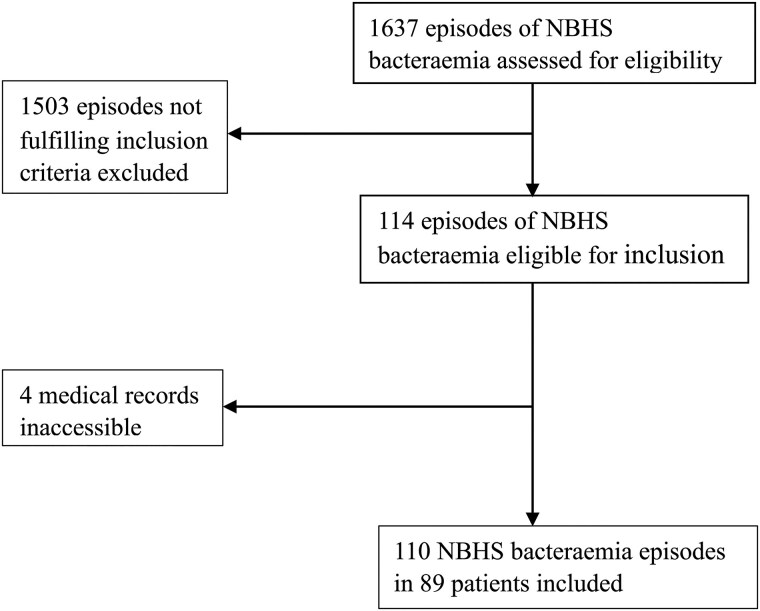
Patient inclusion. The figure presents a flowchart showing the inclusion and exclusion process of non–β-hemolytic streptococcal (NBHS) bacteremia episodes in this study.

Figure 1 presents a flowchart of the inclusion and exclusion process. A total of 1637 episodes of NBHSB were identified and assessed for eligibility. Of these, 110 episodes of NBHSB were identified in 89 patients with HVP. Of the 89 patients, 15 had multiple episodes of NBHSB. In 40 episodes (36%), a diagnosis of definite IE was made. Of the remaining 70 episodes, 69 were classified as possible IE and 1 as rejected IE.

### Baseline and Clinical Characteristics

Table 1 presents the characteristics of NBHSB episodes in patients with HVP. These episodes were divided into episodes where a definite diagnosis of IE was made and episodes that were classified as nondefinite IE. The median age at admission was 80 years (IQR, 69–84) and most patients were male. There were few significant differences between these groups, but *Streptococcus anginosus* bacteremia was significantly more common in nondefinite IE (11% vs 0%, *P* = .049).

**Table 1.** Baseline Characteristics of Cohort Divided into Definite and Nondefinite IE

Variable	HVP and NBHSB (n = 110)	Definite IE (n = 40)	Nondefinite IE (n = 70)	*P* Value
Age, y	80 (69–84)	81 (68–83)	79 (70–84)	.54
Female sex	36 (33)	16 (40)	20 (29)	.29
Charlson comorbidity score				.64
0–1	54 (49)	18 (45)	36 (51)	
2–3	48 (44)	18 (45)	30 (43)	
≥4	8 (7.3)	4 (10)	4 (5.7)	
Risk factors				
Previous IE	19 (17)	10 (25)	9 (13)	.12
Injection drug use	5 (4.6)	4 (10)	1 (1.4)	.06
Permanent IV catheter	1 (0.9)	0 (0)	1 (1.4)	>.99
NBHSB subgroup				
*S mitis*	42 (38)	18 (45)	24 (34)	.31
*S bovis*	21 (19)	6 (15)	15 (21)	.46
*S salivarius*	17 (15)	7 (18)	10 (14)	.79
*S mutans*	15 (14)	7 (18)	8 (11)	.40
*S anginosus*	8 (7.3)	0 (0)	8 (11)	.**049**
*S sanguinis*	7 (6.4)	2 (5.0)	5 (7.1)	>.99
Polymicrobial bacteremia^a^	7 (6.4)	5 (13)	2 (2.9)	.10
Mode of acquisition				
Community	78 (71)	32 (80)	46 (66)	
Health care	27 (25)	8 (20)	19 (27)	
Nosocomial	5 (4.6)	0 (0)	5 (7.1)	.16
Type of valve prosthesis				
Biological	74 (67)	27 (68)	47 (67)	>.99
Mechanical	15 (14)	5 (13)	10 (14)	>.99
TAVI	11 (10)	3 (7.5)	8 (11)	.74
Repair surgery	8 (7.3)	4 (10)	4 (5.7)	.46
Combined	2 (1.8)	1 (2.5)	1 (1.4)	>.99
Location of prosthetic valve				
Aortic	99 (91)	32 (82)	67 (96)	.**03**
Aortic only	94 (85)	31 (78)	63 (90)	
Combined^b^	9 (8.2)	4 (10)	5 (7.1)	
Pulmonary	3 (2.7)	2 (5.0)	1 (1.4)	
Mitral	2 (1.8)	1 (2.5)	1 (1.4)	
Tricuspid	1 (0.9)	1 (2.5)	0 (0)	
Unspecified	1 (0.9)	1 (2.5)	0 (0)	
Focal infection				
Not identified	90 (82)	33 (83)	57 (81)	>.99
Bone/joint	9 (8.2)	5 (13)	4 (5.7)	
Liver/biliary/gastrointestinal	5 (4.6)	0 (0)	5 (7.1)	
Dental	3 (2.7)	2 (5.0)	1 (1.4)	
Pulmonary	2 (1.8)	0 (0)	2 (2.9)	
Abdominal aortic graft	1 (0.9)	0 (0)	1 (1.4)	
Time from surgery, y	4 (2–8)	5 (1–9)	4 (2–8)	.69
Sepsis	21 (19)	8 (20)	13 (19)	>.99
HANDOC score	4 (4–5)	5 (4–5)	4 (3–5)	.07
HANDOC score ≥3	103 (94)	38 (95)	65 (93)	>.99
Highest C-reactive protein^c^	95 (54–144)	101 (65–141)	86 (53–155)	.53
CIED	20 (18)	8 (20)	12 (17)	.80

Categorical variables are reported as No. (%). Nonnormally distributed continuous variables are reported as median (IQR). The Fisher exact test was performed for categorical variables, except for Charlson comorbidity score, for which the Pearson χ^2^ test was conducted. The Mann-Whitney *U* test was conducted for nonnormally distributed continuous variables. Bold indicates *P* < .05.

Abbreviations: CIED, cardiac implantable electronic device; HVP, heart valve prosthesis; IE, infective endocarditis; IV, intravenous; NBHSB, non–β-hemolytic streptococcal bacteremia; TAVI, transcatheter aortic valve implantation.

^a^In the definite IE group, the following microorganisms were identified in the 5 polymicrobial episodes: *Enterococcus faecalis*, *Staphylococcus aureus*, *Staphylococcus hominis*, *Streptococcus bovis group*, and *Streptococcus mitis*. In the nondefinite IE group, the following microorganisms were identified in the 2 polymicrobial episodes: *Enterococcus faecium* and *Klebsiella aerogenes*.

^b^Combined mitral and tricuspid: 4 (3.6), 3 (7.5), 1 (1.4). Combined aortic and pulmonary: 2 (1.8), 0 (0), 2 (2.9). Combined aortic, mitral, and tricuspid: 2 (1.8), 1 (2.5), 1 (1.4). Combined aortic and mitral: 1 (0.9), 0 (0), 1 (1.4).

^c^Within 24 hours from the first positive blood culture result.

Supplementary Table 1 presents the characteristics of the 89 index NBHSB episodes. Table 2 shows the variables included in the Duke criteria of the NBHSB episodes in patients with HVP. The major criterion for blood culture positivity was fulfilled in 35 (88%) episodes with definite IE and 63 (90%) episodes with nondefinite IE (*P* = .69). Only 28 of the 70 episodes (40%) without definite IE were treated with long-course antibiotics as if they had IE. Seven patients underwent cardiac surgery and all had definite IE.

**Table 2.** Diagnostic Criteria According to the Modified Duke/ESC 2015 Criteria of Definite and Nondefinite IE Episodes

Variable	HVP and NBHSB (n = 110)	Definite IE (n = 40)	Nondefinite IE (n = 70)
Pathologic criteria	6 (5.5)	6 (15)	0 (0)
Major criteria			
Blood culture positive for IE	98 (89)^a^	35 (88)	63 (90)
Imaging positive for IE^b^			
Echocardiography	30 (27)	30 (75)	0 (0)
TTE	11 (10)	11 (28)	0 (0)
TOE	28 (25)	27 (68)	0 (0)
PET/CT	8 (7.3)	8 (20)	0 (0)
Cardiac CT	2 (1.8)	2 (5.0)	0 (0)
Minor criteria			
Predisposition	110 (100)	40 (100)	70 (100)
Fever >38 °C	95 (86)	36 (90)	59 (84)
Vascular phenomena	9 (8.2)	7 (18)	2 (2.9)
Immunologic phenomena	1 (0.9)	1 (2.5)	0 (0)
Microbiological evidence	12 (11)	5 (13)	7 (10)
Diagnosis			
Definite IE	40 (36)	40 (100)	0 (0)
Possible IE	69 (63)	0 (0)	69 (99)
Rejected IE	1 (0.9)	0 (0)	1 (1.4)
Treated as IE	68 (62)	40 (100)	28 (40)
Underwent cardiac surgery	7 (6.4)	7 (18)	0 (0)

Variables are reported as No. (%).

Abbreviations: CT, computed tomography; ESC, European Society of Cardiology; HVP, heart valve prosthesis; IE, infective endocarditis; NBHSB, non–β-hemolytic streptococcal bacteremia; PET/CT, positron emission tomography/computed tomography; TOE, transesophageal echocardiography; TTE, transthoracic echocardiography.

^a^In only 8 episodes were >2 blood cultures taken (ie, 3 or 4). In 4 of these episodes, the patient had definite IE; in the other 4 episodes, the patient had nondefinite IE. All of the blood cultures were positive in 7 of these 8 episodes.

^b^TTE: 103 (94), 39 (98), 64 (91). TOE: 92 (84), 35 (88), 57 (81). PET/CT: 18 (16), 8 (20), 10 (14). Cardiac CT: 4 (3.6), 2 (5), 2 (2.9).

### Diagnostic Process

**Figure 2.**
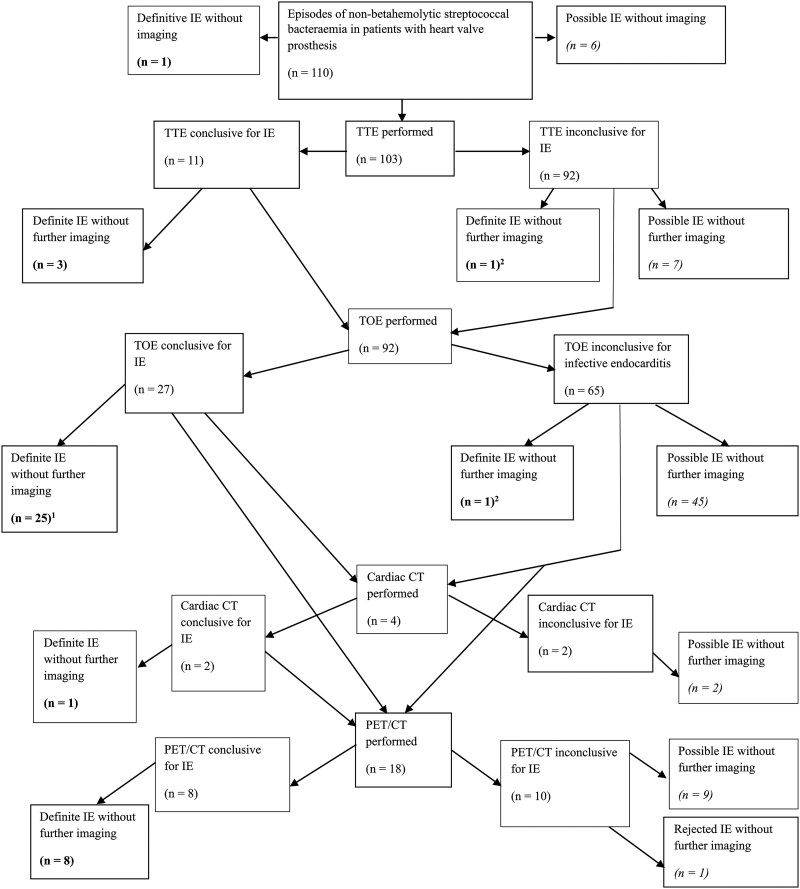
Diagnostic process. The figure presents a flowchart depicting the stepwise diagnostic process for infective endocarditis (IE). ^1^All 27 patients with conclusive TOEs fulfilled the Duke criteria for definite IE. Of these 27 patients, 25 did not undergo imaging beyond TOE. Of the remaining 2 patients, 1 each underwent cardiac CT and PET/CT and is included in the definite cases on the left-hand side after “cardiac CT conclusive for IE” and “PET/CT conclusive for IE.” Each of the 110 patients is included only once as having definite, possible, or rejected IE in the figure. ^2^Two patients with inconclusive echocardiograms were classified as having definite IE. These patients did not fulfill the major criterion of positive imaging. However, they fulfilled 1 major microbiology criterion and 3 minor criteria, leading them to be classified as having definite IE according to the Duke criteria. Abbreviations: CT, computed tomography; PET/CT, positron emission tomography/computed tomography; TOE, transesophageal echocardiography; TTE, transthoracic echocardiography.

Figure 2 presents a flowchart of the diagnostic process for IE in this study. Of the 110 episodes, 1 episode fulfilled the criteria for definite IE without any imaging. In 6 episodes, no imaging was performed. TTE was performed in 103 episodes, and in 92 episodes, the examination did not demonstrate IE. In 92 episodes, a TOE was performed. TOE revealed IE in 27 episodes. All 27 episodes could then be classified as definite IE. In 17 of the 65 episodes in which TOE did not confirm IE, PET/CT was performed. In 7 of these 17 episodes, the scan led to a definite IE diagnosis. In 3 of the 65 episodes in which TOE did not demonstrate IE, cardiac CT was performed. One of the 3 cardiac CT scans showed signs of IE.

### Treatment and Outcome

**Figure 3.**
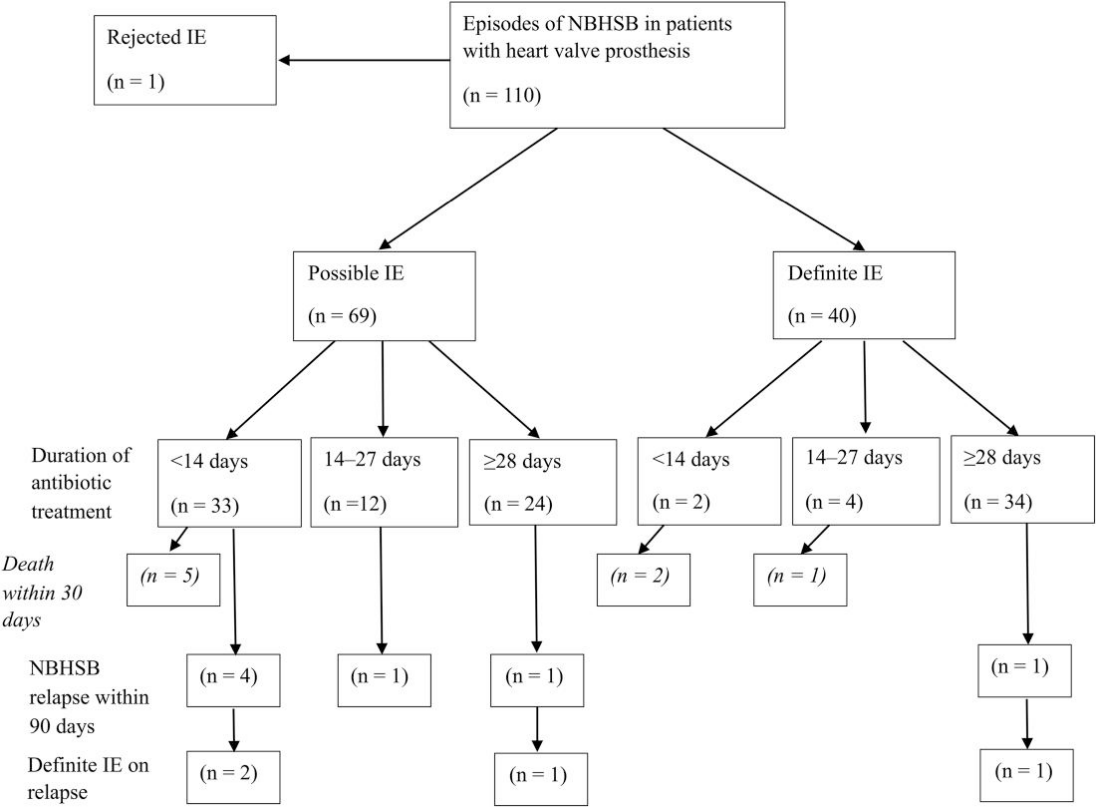
Duration of antibiotic treatment and relapse of non–β-hemolytic streptococcal bacteremia (NBHSB) in patients with possible vs definite infective endocarditis (IE). The figure depicts a flowchart of relapsing NBHSB episodes within 90 days after completion of antibiotic treatment depending on the duration of antibiotic treatment. Patients who were diagnosed with definite IE on relapse are presented. Deaths within 30 days after a positive blood culture result are also presented.

In Figure 3, a flowchart outlines the duration of antibiotic treatment in the episodes of NBHSB that fulfilled the criteria for possible or definite IE. Furthermore, Figure 3 shows the NBHSB relapses depending on the duration of antibiotic therapy. Deaths that occurred within 30 days after a positive blood culture result are presented. There were 6 (8.7%) episodes of recurrent NBHSB within 90 days in patients with possible IE. In the majority of the 40 (85%) episodes of definite IE, the duration of antibiotic treatment was at least 28 days. There was only 1 episode of NBHSB relapse in patients with definite IE, and this patient was diagnosed with definite IE again at the time of the relapse.

In Supplementary Table 2, all 7 NBHSB relapses are presented. Four of the patients with NBHSB relapse were diagnosed with definite IE on relapse. The time until relapse after completion of intravenous antibiotic therapy varied from 11 to 72 days. None of the patients with NBHSB relapse underwent cardiac surgery during the index episode of NBHSB.

## DISCUSSION

In this study, 36% of the patients with NBHSB and HVP had definite IE, and this proportion might be falsely low, considering that not all patients underwent detailed imaging. Thus, the risk for IE is high in this patient population. Previous studies have not addressed the absolute risk for IE in this group, although several studies have identified HVP as a risk factor for IE in NBHSB. For example, Chamat-Hedemand et al found HVP to be a risk factor for IE in patients with streptococcal bloodstream infections, with an odds ratio of 3.8 [6]. Similarly, Seo et al found that HVP is an independent risk factor for IE in streptococcal bloodstream infections [5]. Sunnerhagen et al included HVP and native valve disease in their variable “preexisting heart valve disease,” which they found to be a risk factor for IE in NBHSB [11].

The majority of the 40 patients with definite IE were treated for IE with long-course antibiotic treatment. Even though only 7 of the 40 patients underwent cardiac surgery and some patients did not receive aminoglycosides, there was only 1 patient who had a relapse of NBHSB (Supplementary Table 2). This patient was not subjected to surgery and did not receive the recommended combination with an aminoglycoside. Furthermore, this patient had injection drug use, suggesting that the relapse of bacteremia might have been a reinfection. Supporting this speculation, the patient had an additional episode of NBHSB with another species in between the episodes of definite IE. The low relapse rate after completion of the recommended antibiotic duration of 4 to 6 weeks suggests that it might be possible to shorten the duration of treatment without compromising its efficacy. Such a possibility is currently investigated in the POET Ⅱ trial [20].

We could not identify many factors associated with IE in patients with NBHSB and HVP. Interestingly, there was no significant difference in the proportion of patients at high risk for IE according to the HANDOC score between patients with definite and nondefinite IE. A similarity with the HANDOC study and other studies in the field was that we found *Streptococcus anginosus* to be less common in patients with definite IE [5, 6, 11]. It is possible that the risk of IE differs among the different species within the groups, especially within the *Streptococcus bovis* group. However, since species determination within the *S bovis* group is difficult, we chose not to report the species or subspecies.

In patients who received a diagnosis of possible IE, a minority received treatment as if they had IE. Many of the patients who received short-course treatment had not undergone a thorough workup for IE. We find it likely that some of the patients who received short-course treatment and had a relapse would have been found to have IE with a thorough workup, possibly including TOE and PET/CT. In favor of this speculation is the observation that 2 of the 4 episodes of NBHSB relapse in patients who received short-course treatment had definite IE on relapse. We therefore believe that a more thorough investigation of patients with NBHSB and HVP could decrease the risk for relapse. However, it can also be argued that many patients did not experience relapse despite short-course treatment; thus, we believe that not all patients with NBHSB and HVP need treatment for IE. For patients who receive a short course of treatment, the patient and the health care provider should be aware of the risk for relapse and ensure appropriate follow-up to rapidly detect a renewed episode. Importantly, relapse occurred as early as 11 days after completion of therapy, but in 1 case relapse was detected after >2 months after completion of treatment (Supplementary Table 2).

The main strength of our study is that it was performed in a large population-based cohort. Considering that there is only 1 microbiological laboratory serving all the hospitals in the region of Skåne, we feel certain that all episodes of NBHSB, including relapses, were identified. Moreover, the population-based design of our study is a strength, further helping to avoid selection bias and increase the generalizability of the results. We could study the medical records of all patients and, through this procedure, make a precise classification according to the Duke criteria. This approach is much more precise than relying on *ICD* codes as some studies in the field have done [12].

A limitation of our study is that not all patients underwent the recommended TTE and TOE; thus, we might have missed the diagnosis of definite IE in some patients. Cardiac CT or PET/CT was performed in few patients, increasing the likelihood of a missed diagnosis. Furthermore, due to deaths occurring within 90 days after completion of the antibiotic therapy, we might have missed some recurrent episodes of NBHSB. Although we consider the cohort to be large in comparison with similar studies, it is not large enough to investigate more subtle differences regarding relapses, and the retrospective design subjects the study to bias due to missing data and loss of follow-up. Another weakness is that we cannot be certain whether the recurrent episodes within 90 days were true relapses or rather reinfections with the same species. The patient who was injecting drugs and had 2 episodes of definite IE is likely to have had a reinfection rather than a relapse.

Despite these limitations, our results imply that patients with NBHSB and HVP have a significant risk for IE. We suggest that the finding of NBHSB in patients with HVP should warrant TOE, and in patients with a negative TOE, PET/CT or cardiac CT could be considered. However, the decision on treatment length will ultimately be a holistic one taking into account several clinical features and patient preferences.

## Supplementary Material

ofaf242_Supplementary_Data

## Supplementary Data

Supplementary materials are available at *Open Forum Infectious Diseases* online. Consisting of data provided by the authors to benefit the reader, the posted materials are not copyedited and are the sole responsibility of the authors, so questions or comments should be addressed to the corresponding author.
