# Supplementary material for: Non–β-hemolytic Streptococcal Bacteremia in Patients With Heart Valve Prosthesis—Is It Always Infective Endocarditis?
Source: Open Forum Infect Dis. 2025 Apr 18;12(5):ofaf242. doi: 10.1093/ofid/ofaf242 (PMC12056612; doi:10.1093/ofid/ofaf242)
Supplement: ofaf242_Supplementary_Data [file ofaf242_supplementary_data.docx]

Supplementary Material

Table 1. Baseline characteristics of definite IE and non-definite IE episodes with the 89 index NBHSB episodes included.

| Variables | HVP and index NBHSB  n = 89 | Definite IE  n = 29 | Non-definite IE  n = 60 | P value |
| --- | --- | --- | --- | --- |
| Age (years) | 78 (70 – 84) | 80 (69 – 82) | 78 (70 – 85) | .50 |
| Female sex | 29 (33) | 11 (38) | 18 (30) | .48 |
| Charlson comorbidity score  0-1  2-3  ≥4 | 46 (52)  36 (40)  7 (7.9) | 15 (52)  10 (34)  4 (14) | 31 (52)  26 (43)  3 (5.0) | .32 |
| Risk factors  Previous IE  Injection drug use  Permanent IV catheter | 7 (7.9)  2 (2.3)  1 (1.1) | 4 (14)  2 (6.9)  0 (0) | 3 (5.0)  0 (0)  1 (1.7) | .21  .10  1.0 |
| NBHS subgroup  S*. mitis*  *S. bovis*  *S. salivarius*  *S. mutans*  *S. anginosus*  *S. sanguinis* | 32 (36)  17 (19)  13 (15)  14 (16)  8 (9.0)  5 (5.6) | 13 (45)  4 (14)  5 (17)  6 (21)  0 (0)  1 (3.5) | 19 (32)  13 (22)  8 (13)  8 (13)  8 (13)  4 (6.7) | .25  .57  .75  .37  .05  1.0 |
| Polymicrobial bacteraemia^1^ | 6 (6.7) | 4 (14) | 2 (3.3) | .09 |
| Mode of acquisition  Community  Healthcare  Nosocomial | 70 (79)  14 (16)  5 (5.6) | 28 (97)  1 (3.5)  0 (0) | 42 (70)  13 (22)  5 (8.3) | .17 |
| Type of valve prosthesis  Biological  Mechanical  TAVI  Repair surgery  Combined | 61 (69)  15 (17)  8 (9.0)  4 (4.5)  1 (1.1) | 20 (69)  5 (17)  2 (6.9)  2 (6.9)  0 (0) | 41 (68)  10 (17)  6 (10)  2 (3.3)  1 (1.7) | 1.0  1.0  1.0  .59  1.0 |
| Location of prosthetic valve  Aortic  Aortic only  Combined^2^  Pulmonary  Mitral  Tricuspid  Unspecified | 83 (93)  79 (89)  4 (4.5)  2 (2.3)  2 (2.3)  1 (1.1)  1 (1.1) | 25 (86)  24 (83)  1 (3.5)  1 (3.5)  1 (3.5)  1 (3.5)  1 (3.5) | 58 (97)  55 (92)  3 (5.0)  1 (1.7)  1 (1.7)  0 (0)  0 (0) | .09 |
| Focal infection  Not identified  Bone/joint  Liver/biliary/gastrointestinal  Dental  Pulmonary  Abdominal aortic graft | 71 (80)  7 (7.9)  5 (5.6)  3 (3.4)  2 (2.3)  1 (1.1) | 24 (83)  3 (10)  0 (0)  2 (6.9)  0 (0)  0 (0) | 47 (78)  4 (6.7)  5 (8.3)  1 (1.7)  2 (3.3)  1 (1.7) | .78 |
| Time from surgery (years) | 4 (2 – 8) | 4 (1 – 8) | 4 (2 – 9) | .63 |
| Sepsis | 18 (20) | 5 (17) | 13 (22) | .78 |
| HANDOC score | 5 (4 – 5) | 5 (4 – 5) | 4 (3 – 5) | **.045** |
| HANDOC score ≥ 3 | 83 (93) | 27 (93) | 56 (93) | 1.0 |
| Highest CRP^3^ | 95 (54 – 159) | 95 (46 – 144) | 92 (55 – 160) | .96 |
| CIED | 15 (17) | 5 (17) | 10 (17) | 1.0 |

Categorical variables are reported as N (%). Non-normally distributed continuous variables are reported as median (interquartile range). The Fisher’s exact test was performed for categorical variables, except for the variable Charlson comorbidity score for which the Pearson’s chi-squared test was conducted. The Mann-Whitney U-test was conducted for non-normally distributed continuous variables. Abbreviations: IE, infective endocarditis; HVP, heart valve prosthesis; NBHSB, non-beta-haemolytic streptococcal bacteraemia; TAVI, transcatheter aortic valve implantation; CIED, cardiac implantable electronic device; IV, intravenous.^1^In the group definite IE, the following microorganisms were identified in the four polymicrobial episodes, respectively: *Enterococcus faecalis, Staphylococcus aureus, Staphylococcus hominis, and Streptococcus mitis*. In the group non-definite IE, the following microorganisms were identified in the two polymicrobial episodes, respectively: *Enterococcus faecium, and Klebsiella aerogenes*. ^2^Combined mitral and tricuspid: 1 (1.1); 1 (3.4); 0 (0), combined aortic and pulmonary: 1 (1.1); 0 (0); 1 (1.7), combined aortic, mitral, and tricuspid: 1 (1.1); 0 (0); 1 (1.7), combined aortic and mitral: 1 (1.1); 0 (0); 1 (1.7). ^3^Within 24 hours from the first positive blood culture.

Table 2. Relapses of non-beta-haemolytic streptococcal bacteraemia in patients with heart valve prosthesis.

| Age (years) | Sex | Type of valve prosthesis | Index episode | NBHS subspecies | TTE/TOE (0=neg, 1=pos) | Days until relapse | Outcome on relapse | Days of beta-lactam therapy | Days of aminoglycoside therapy | Cardiac surgery |
| --- | --- | --- | --- | --- | --- | --- | --- | --- | --- | --- |
| 86 | Male | Biological | Possible IE | *S. mitis* | 0/0 | 11 | NBHSB | 9 | 0 | No |
| 87 | Female | Biological | Possible IE | *S. mutans* | 0/0 | 66 | Definite IE | 13 | 0 | No |
| 33 | Male | Valve repair | Possible IE | *S. sanguinis* | 0/0 | 19 | NBHSB | 14 | 0 | No |
| 82 | Female | Biological | Possible IE | *S. bovis* | 0/0 | 19 | Definite IE | 10 | 2 | No |
| 81 | Male | TAVI | Possible IE | *S. bovis*^1^ | 0/0 | 20 | NBHSB | 8 | 0 | No |
| 81 | Male | TAVI | Possible IE | *S. bovis*^1^ | 0/0 | 53 | Definite IE | 32 | 14 | No |
| 42 | Male | Valve repair | Definite IE | *S. mitis* | 0/1 | 72 | Definite IE | 42 | 0 | No |

Abbreviations: NBHS, non-beta-haemolytic streptococci; TTE, transthoracic echocardiography; TOE, transoesophageal echocardiography; TAVI, transcatheter aortic valve implantation; IE, infective endocarditis; NBHSB, non-beta-haemolytic streptococcal bacteremia. ^1^The three strains of S. bovis in the three episodes of NBHSB in this patient (index episode and two relapses) were found to be clonally related in another study (21).


**References:**

21. Öberg J, Rasmussen M, Buchwald P, et al. *Streptococcus bovis*-bacteremia: subspecies distribution and association with colorectal cancer: a retrospective cohort study. Epidemiol Infect. 2021 Nov 26;150:e8.
